# Common Variants in TRDN and CALM1 Are Associated with Risk of Sudden Cardiac Death in Chronic Heart Failure Patients in Chinese Han Population

**DOI:** 10.1371/journal.pone.0132459

**Published:** 2015-07-21

**Authors:** Zhouying Liu, Xiaoyan Liu, Haiyun Yu, Juanhui Pei, Yinhui Zhang, Jing Gong, Jielin Pu

**Affiliations:** State Key Laboratory of Cardiovascular Disease, Physiology and Pathophysiology Laboratory, Fuwai Hospital, National Center for Cardiovascular Diseases, Chinese Academy of Medical Sciences and Peking Union Medical College, 167 Bei-Li-Shi Road, Xi-Cheng District, Beijing, 100037, P. R. China; University of Newcastle, AUSTRALIA

## Abstract

**Background:**

Recent studies suggest that variants in two calcium handling genes (RyR2 and CASQ2) associated with sudden cardiac death (SCD) and non-sudden cardiac death (NSCD) in subjects with heart failure and coronary artery disease, respectively. The purpose of this study was to identify other calcium handling genes associated with SCD in the long-term of chronic heart failure (CHF) in Chinese Han population.

**Methods and Results:**

We investigated 20 SNPs representing 10 genes that regulated calcium handling in 1429 patients with CHF, and the genetic association with SCD and all-cause death was analysed. During a median follow-up period of 63 months, 538 patients (37.65%) died from CHF, of whom 185 (34.38%) had SCD and the others were NSCD. SNPs that pass a P value cut-off of 0.0025 were considered as significant. We found that patients carrying the CC genotype of rs3814843 on CALM1 gene had greater risks of SCD (HR 5.542, 95% CI 2.054–14.948, P = .001) and all cause death (HR 3.484, 95% CI 1.651–7.350, P = .001). After adjusting for other risk factors, significant associations remained. Moreover, patients carrying G allele of rs361508 on TRDN gene also had increased risk of SCD.

**Conclusions:**

Common variants in TRDN and CALM1 are associated with increased risk of SCD in patients with CHF. These findings provide further evidence for association of variants in calcium handling regulating proteins and SCD in chronic heart failure.

## Introduction

Chronic Heart failure (CHF) afflicts 4 million people in China, with increased incidence and prevalence in aged subjects[1]. Sudden cardiac death (SCD) is one of the main causes of death in patients with CHF and accounts for one-third of the total CHF deaths [2]. In most cases, SCD is caused by lethal ventricular arrhythmia, such as ventricular tachycardia (VT) and ventricular fibrillation (VF). Therefore, the risk stratification has been recognized as a pivotal step towards reduced mortality. Several aspects have been recognized as predictors of SCD, such as electrophysiological parameters, hemodynamic status, and biomarkers, but the specificity and sensitivity are not satisfactory so far[3].

Ca^2+^ is a central mediator of excitation contraction coupling of the human heart. Ca^2+^ enters into the cytoplasm by L-type Ca^2+^ channels (LTCC) following membrane depolarization, which triggers a large amount of Ca^2+^ release from sarcoplasmic reticulum (SR) through RyR2 (SR Ca^2+^ release channel). Following contraction, cytoplasmic Ca^2+^ is taken up into SR by Ca^2+^ ATPase (SERCA2) or extruded outside of the cell by Na- Ca^2+^ exchanger [4]. Moreover, Ca^2+^ plays crucial role in lethal arrhythmias. It is generally accepted that Ca^2+^ is the key component in the initiation of delayed after-depolarization (DAD) and early after-depolarization (EAD), which can trigger lethal arrhythmia[5]. Function of crucial channels (LTCC, RyR2 and SERCA2) is regulated by regulatory proteins and adrenergic receptor signaling cascade, such as calsequestrin 2 (CASQ2), calmodulin (CALM1), phospholamban (PLN), protein kinase A (PRKACG), and Ca^2+^ **/**calmodulin-dependent protein kinases IIδ(CAMK2D).

Previous studies have shown that variation in genes could be associated with SCD[6], of them, most important were common genetic variant in RyR2, CASQ2, ATP2A2 and NOS1AP genes[7–11]. We hypothesized that variations in genes that regulated channels could be associated with SCD in HF patients. Therefore, we conducted a candidate-gene sequencing to study the association between SNPs and SCD in HF patients, among genes known to regulate LTCC, RyR2 and SERCA2.

## Methods

### 2.1 Study population

We conducted an ongoing prospective study of patients with CHF from Fu Wai Cardiovascular Hospital from 2005 to 2009. Enrolment criteria included: CHF caused by dilated cardiomyopathy (DCM) or ischemic cardiomyopathy (ICM); in NYHA (New York Heart Association) functional class II–IV despite optimized medical therapy; and LVEF ≤45% in DCM and ≤50% in ICM. DCM was diagnosed according to the guidelines for the study of familial DCM [12]. ICM was defined as ≥70% luminal stenosis of at least one major coronary artery diagnosed by coronary angiography and a history of myocardial infarction >3 months before enrolment. All cases were excluded if they had malignant tumors, severe liver and kidney dysfunctions, or other uncontrollable system diseases, pregnancy, or unwillingness to participate in the study. Consequently, 1429 patients (484 DCM and 945 with ICM) were available for analysis.

The investigation adhered to the principles outlined in the Declaration of Helsinki and was approved by the Ethics Committee of Fu Wai Cardiovascular Hospital (Beijing, China). All subjects who participated in the study provided written informed consent and reported themselves as being of Chinese Han nationality.

### 2.2 End point assessment

The study population was followed up for a median period of 63 months until August 2014. A standard HF questionnaire was completed during regular outpatient clinics or by telephone contact. The end points included all cause death, SCD and non-SCD (NSCD). SCD was defined operationally as a witnessed natural death attributable to cardiac causes heralded by an abrupt loss of consciousness within 1 h of onset of acute symptoms, or as an unexpected death of someone seen in a stable medical condition <24 h previously with no evidence of a non-cardiac cause [3].

### 2.3 DNA Extraction and Genotyping

Genome DNA was extracted from peripheral blood leukocytes using standard method and stored at −70°C after determination of absorbance at 260 nm followed by Picogreen analysis (Molecular Probes, Eugene, Oegon, USA)[13].

Subsequently, SNP selection was performed in mid-2013 based on 1000 Genomes and the HapMap data release No. 28/phase Ⅱ August 2010, applying the following criteria: genes associated with calcium regulation including enzymes of AC-cAMP-PKA-CAMKⅡ cascade and regulators of RyR2, L-type calcium channel and Ca^2+-^ATPase, CHB population, and a minor allele frequency (MAF) >0.01. Linkage disequilibriums (LDs) were calculated using pairwise tagging only with a cutoff of r^2^>0.8. We used the candidate approach and undertook a search of variants in the potentially functional region, including exons, promoter region, upstream and downstream regions. Finally, 71 SNPs representing 20 genes of interest were selected for first genotyping analysis based on above criterions including three reported SNPs (rs17500488, rs3010396, and rs7366407) associated with SCD[8]. A list of selected SNPs is shown in S1 Table. Polymerase chain reactions were performed in 384 CHF patients, and then 20 SNPs representing 10 genes were selected for subsequent genotyping based on interesting associations in analysis of first 384 patients in the entire study cohort (Table 1).

**Table 1:** Candidate genes and list of selected SNPs.

Gene	SNP	Position	MAF in CHB	Function Prediction
**PRKACG**	rs3730386	missense	0.397	nsSNP
**CAMKⅡD**	rs10033516	promoter	0.47	TFBS
**CALM1**	rs3814847	promoter	0.167	TFBS
	rs3814843	3' UTR	0.116	TFBS
	rs1058903	3' UTR	0.093	miRNA binding site
	rs5871	3' UTR	0.402	miRNA binding site
**PSEN2**	rs8383	3' UTR	0.34	miRNA binding site
	rs1295645	5' UTR	0.283	miRNA binding site
**CASQ2**	rs17500488	intron	0.155	TFBS
	rs3010396	intron	0.422	
	rs7366407	near gene-5'	0.356	
**ASPH**	rs4507756	3' UTR	0.082	miRNA binding site
	rs7003147	promoter	0.227	miRNA binding site
	rs6759	exon-synon	0.349	miRNA binding site
**TRDN**	rs9490809	missense	0.061	Splicing regulation
	rs361508	3' UTR	0.395	miRNA binding site
	rs6902416	missense	0.078	miRNA binding site
**CD38**	rs1800051	exon-synon	0.195	Splicing regulation
**PPP1CA**	rs77472930	exon-missense	0.103	miRNA binding site
**ADRBK1**	rs12791853	near gene-5'	0.103	TFBS

Function Prediction is completed by an online database (SNP Function Prediction, http://snpinfo.niehs.nih.gov/snpinfo/snpfunc.htm).

Primers were designed with a combination of previous articles and AssayDesigner3.1 (S2 Table). Genotyping was performed according to manufacturer’s instruction using MALDI-TOF MS genomics platform, Sequenom MassARRAY system (USA).

### 2.4 Statistical Analysis

Continuous values were expressed as mean±SD and categorical variables as numbers (%). Hardy-Weinberg equilibrium (HWE) of alleles was evaluated with the use of the chi-square test with one degree of freedom across whole cohort. The genotype frequencies were compared between patients with and without the ending point. Person-months of the follow-up period were from the date of enrollment to the end of August 2014. Survival analysis was performed on all variants. In addition to the genotype, univariate analysis and multivariate Cox proportional hazards models were performed to estimate the effect of genotype on survival. Univariate predictors of the genotype and SCD mortality with a P value of <0.0025 were included into the multivariate models. Kaplan-Meier curves with the use of the log-rank test according to the presence or absence of the allele was also analyzed. Three models including dominant model, recessive model an additive model were constructed for each variant.

## Results

### 3.1 Clinical characteristics and genotyping

The clinical characteristics of CHF patients are shown in S3 Table. In brief, a total of 1429 CHF patients (1129 males; 79.01%) with a mean age of 63.76±12.27 years were included. The underlying aetiologies of CHF were ICM (945 patients; 66.13%) with a mean age of 65.56±10.57 years and DCM (484 patients; 33.87%) with a mean age of 60.21±14.44 years.

Hardy-Weinberg equilibrium (HWE) of all SNPs was evaluated with the use of the chi-square test with one degree of freedom. All SNPs were in HWE except for two SNPs (rs28730709, rs7948284), see S4 Table.

### 3.2 Associations between variants and SCD in patients with CHF

Over a median follow-up of 63 months in 1,429 patients with CHF, 538 patients (37.65%) died from CHF (203 in DCM and 335 in ICM), of whom 185 (34.38%) had SCD (64 in DCM and 121 in ICM) and the others were NSCD. We analyzed the end points with the all variants using a survival Cox regression analysis in the CHF population (S5 Table).

Only rs3814843 was considered as significantly associated with SCD and all cause death (passed the significant cut-off point at 0.05/20 = 0.0025 (P = 0.05/20 SNPs) level). As demonstrated in Table 2, patients carrying the CC genotype of rs3814843 had increased risks of SCD (recessive model: HR 5.542, 95% CI 2.054–14.948; P = 0.001) and all cause death (recessive model: HR 3.484, 95% CI 1.651–7.350; P = 0.001). However, there were two SNPs (rs361508, rs1800051) that might also be associated, which passed a 0.05 cut-off instead of 0.0025. So patients carrying the G allele of rs361508 might have increased risks of SCD (additive model, HR 1.247, 95% CI 1.021–1.524; P = 0.03). Patients carrying the C allele of rs1800051 might also have increased risks of all cause death (dominant model: HR 1.204, 95% CI 1.010–1.434; P = 0.039) and SCD (dominant model: HR 1.391, 95% CI 1.036–1.867; P = 0.028 and additive model: HR 1.342, 95% CI 1.058–1.702; P = 0.015).

**Table 2:** SNPs Significantly Associated with SCD and All Cause Death.

SNP/ Ending Point	Model	Cox Regression	
		P Value/HR(95%CI)	Adjusted P Value/HR(95%CI)
**rs361508**/SCD	DOM	0.050/1.379(1.001 1.900)	0.033**†/**1.576(1.037 2.396)
	REC	0.121/1.313(.931 1.852)	0.098/1.444(.935 2.230)
	ADDITIVE	0.030**†/**1.247(1.021 1.524)	0.002**‡/**1.329(1.026 1.721)
**rs361508/**NSCD	DOM	0.921/1.011(.812 1.259)	0.719/1.049(.809 1.360)
	REC	0.18/1.192(.922 1.540)	0.401/1.144(.835 1.567)
	ADDITIVE	0.426/1.061(.917 1.228)	0.486/1.064(.893 1.268)
**rs361508/**ACD	DOM	0.217/1.120(.935 1.342)	0.136/1.182(.949 1.474)
	REC	0.046**†/**1.233(1.004 1.514)	0.107/1.233(.956 1.590)
	ADDITIVE	0.055/1.122(.997 1.263)	0.059/1.149(.995 1.327)
**rs3814843/**SCD	DOM	0.245/1.247(.859 1.809)	0.24/1.318(.831 2.090)
	REC	0.001**‡/**5.542(2.054 14.948)	0.001**‡/**7.466(2.604 21.403)
	ADDITIVE	0.001**‡/**2.372(1.442 3.900)	0.001**‡/**2.717(1.598 4.619)
**rs3814843/**NSCD	DOM	0.789/.961(.716 1.289)	0.853/.968(.686 1.366)
	REC	0.145/2.331(.747 7.269)	0.068/2.957(.924 9.461)
	ADDITIVE	0.988/.998(.754 1.320)	0.882/1.025(.741 1.418)
**rs3814843/**ACD	DOM	0.641/1.056(.839 1.330)	0.621/1.072(.814 1.412)
	REC	0.001**‡/**3.484(1.651 7.350)	0.001**‡/**4.501(2.078 9.749)
	ADDITIVE	0.001**‡/**1.866(1.284 2.712)	0.001**‡/**2.050(1.389 3.023)
**rs1800051/**SCD	DOM	0.028**†/**1.391(1.036 1.867)	0.021**†/**1.553(1.068 2.259)
	REC	0.111/1.642(.893 3.020)	0.471/1.397 (.563 3.465)
	ADDITIVE	0.015**†/**1.342(1.058 1.702)	0.026**†/**1.422(1.043 1.937)
**rs1800051/**NSCD	DOM	0.337/1.113(.894 1.386)	0.182/1.193(.920 1.547)
	REC	0.085/.518(.245 1.094)	0.557/.783(.346 1.771)
	ADDITIVE	0.825/1.021(.847 1.232)	0.330/1.118(.893 1.399)
**rs1800051/**ACD	DOM	0.039**†/**1.204(1.010 1.434)	0.016**†/**1.299(1.050 1.608)
	REC	0.628/.890(.556 1.424)	0.936/.975(.532 1.788)
	ADDITIVE	0.109/1.128(.973 1.306)	0.039**†/**1.211(1.010 1.452)

SNP, single nucleotide polymorphism; DOM, dominant genetic model; REC, recessive genetic model; ADDITIVE, additive genetic model; SCD, sudden cardiac death; NSCD, non- sudden cardiac death; ACD, all cause death. Model 1: unadjusted model. Model 2: model 1+age, LVEF, and other factors, including New York Heart Association Functional class, hypertension, diabetes mellitus, etc.

**†**P<0.05, possibly significant associated.

**‡**P<0.0025, significant associated. Since we have 20 SNPs, we choose the significant cut-off point at 0.05/20 = 0.0025 level.

Then, age, other factors, including NHYA functional class, hypertension, diabetes mellitus, hyperlipidemia, LVEF, LVEDD and survived episodes of sustained VT/VF were included in the multivariate models according to univariate analysis. After adjustment for these factors, CC genotype of rs3814843 was still associated with increased risks of SCD (recessive model: HR 7.466, 95% CI 2.604–21.403; and additive model: HR 2.717, 95% CI 1.598 4.619; P = 0.001) and all cause death (recessive model: HR 4.501, 95% CI 2.078–9.749; and additive model: HR 2.050, 95% CI 1.389 3.023; P = 0.001). What’s more, rs361508 became significantly associated with SCD (Table 2). The results demonstrated that G allele of rs361508 was associated with increased risks of SCD in additive model (HR 1.329, 95% CI 1.026–1.721; P = 0.002). However, rs1800051 just passed a 0.05 cut-off instead of 0.0025 (see Table 2. Association with SCD: P = 0.021; association with all cause death: P = 0.016) in an additive model. Considering it passed the traditional significance cut-off (0.05), whether rs1800051 may pass 0.0025 significance cut-off with more patients or in other population deserves further study.

These results suggest that the G allele of rs361508 on the TRDN gene, and CC genotype of rs3814843 on the CALM1 gene are risk factors for SCD. In addition, CC genotype of rs3814843 is also a risk factor for all cause death. However, C allele of rs1800051 on CD38 gene might be risk factor of SCD and all cause death,

A previous study has revealed 3 SNPs associated with SCD in patients with coronary artery disease: CASQ2 region (rs17500488, rs3010396, and rs7366407)[8]. However, such association in white, non-Hispanic population was not confirmed in our Chinese Han population (S5 Table).

**Fig 1:**
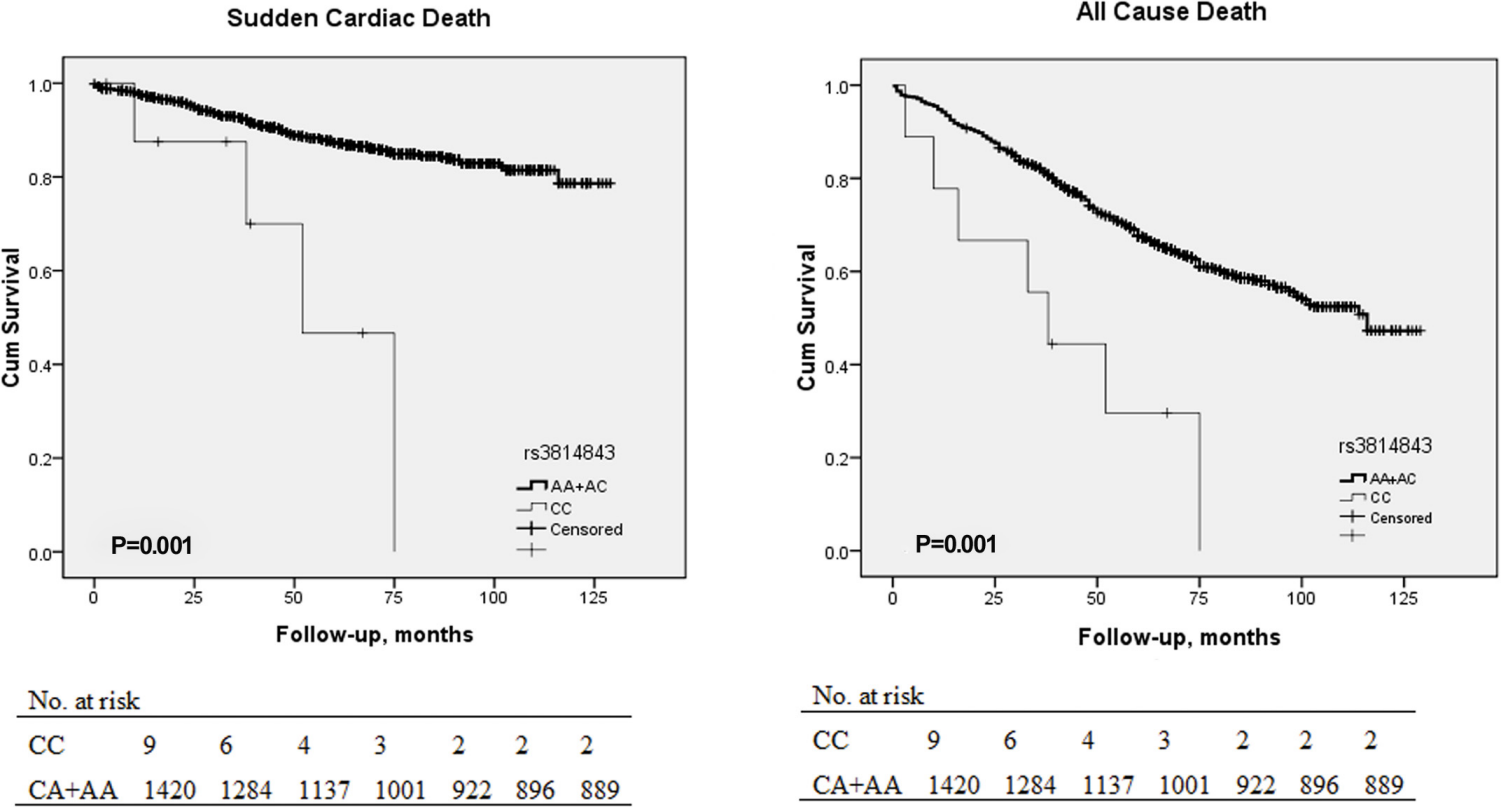
Kaplan-Meier curves in CHF patients according to the presence of CC genotype of rs3814843. Each censored case is marked with a cross mark. Patients carrying the CC genotype of rs3814843 were more susceptible to SCD and all-cause death (Log rank test: P = 0.001, P = 0.001, respectively). The Table at the bottom indicates the number of patients with CHF at risk for every 20 months (the last period was 24 months) of follow-up.

**Fig 2:**
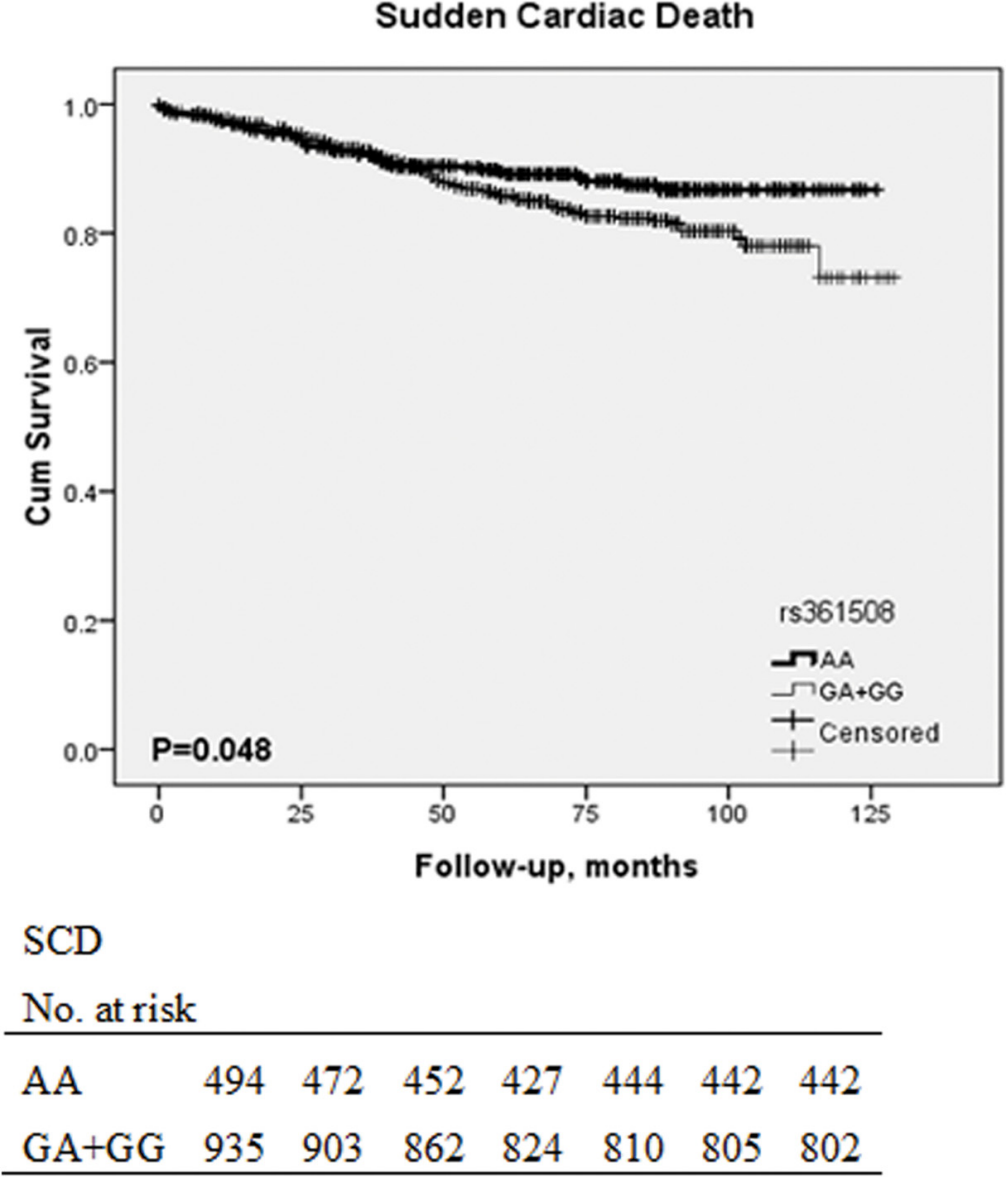
Kaplan-Meier curves in CHF patients according to the presence of G allele of rs361508. Each censored case is marked with a cross mark. Patients carrying the G allele of rs361508 were more susceptible to SCD than patients not carrying (Log rank test: P = 0.048). The Table at the bottom indicates the number of patients with CHF at risk for every 20 months (the last period was 24 months) of follow-up.

When we created Kaplan–Meier curves using the log-rank test for time to probability of survival according to the presence or absence of the CC genotype of rs3814843, the association with SCD (P = 0.001, Fig 1) and all cause death (P = 0.001, Fig 1) remains significant. On the other hand, G allele of rs361508 is not significantly associated with SCD (P = 0.048, Fig 2). In the same way, C allele of rs180005 is also not significantly associated with all cause death (P = 0.038, S4 Table) and SCD (P = 0.027, S4 Table).

## Discussions

In the present study, we observed significant associations between DNA variants located in functional regions of TRDN and CALM1 genes and risk of SCD in subjects with CHF in a Chinese Han population. All of them are involved in calcium signaling. TRDN, CALM1 are of special interest because of their known involvement in the primary arrhythmias. The minor G allele of rs361508 and CC genotype of rs3814843 are independent predictors of SCD. In addition, CC genotype of rs3814843 is also an independent predictor of all cause death.

Westaway et al reported three CASQ2 (a RyR2 regulator) SNPs (rs17500488, rs3010396, rs7366407) associated with SCD in patients with ICM in white, non-Hispanic population [8]. However, the present study showed that their associations with either SCD or all cause death were not observed in Chinese Han populations. This result might suggest that differences in genetic architecture between American and Chinese Han population.

The underlying mechanisms by which variants increases SCD risk have not been fully elucidated. TRDN encodes the sarcoplasmic reticulum anchoring protein tradin in the calcium release complex and involves in anchoring calsequestrin (Gene: CASQ2) to the junctional sarcoplasmic reticulum and allowing its functional coupling with RyR2[14]. CALM1 encodes the cytoplasmic calcium-binding protein calmodulin. Both of them are regulators of RyR2[15] and mutations in both triadin and calmodulin have been associated with catecholaminergic polymorphic ventricular tachycardia, a rare familial arrhythmogenic disorder characterized by maligant ventricular arrhythmias and increased risk of SCD[16, 17]. In addition, Ran et al reported that the one RyR2 SNP (A allele of rs3766871) increased the risk of SCD while another SNP (A allele of rs790896) reduced the risk of SCD in patients with CHF[7]. The A allele of rs3766871 is also a risk factor for ventricular arrhythmias. Westaway et al reported three CASQ2 SNPs (rs17500488, rs3010396, and rs7366407) associated with SCD in patients with ICM[8]. All these results revealed that variants in RyR2 and its regulators play an essential role in the occurrence of SCD, which in agreement with previous hypothesis[18, 19].

Perturbations of ion channels (mainly L-type channel, SERCA2A, RyR2) and Ca^2+^ regulatory machinery impair cellular calcium handling and can initiate delayed after-depolarizations (DADs) and early after-depolarizations (EADs), which in turn trigger lethal arrhythmia probably leading to SCD[18]. Although two recent studies screened for association between SCD and SNPs on ion channel encoding genes in HF population[7, 10], SNPs of ion channel regulators have not been screened. Our results demonstrated that variants of these regulatory proteins also associate with SCD.

Although the specific functional roles of these SNPs have not been investigated, the above studies suggested several possible cellular mechanisms. Both rs361508 and rs3814843 located in 3’UTR regions and were predicted as the potential miRNA binding site. Therefore, one hypothesis might be that SNPs decrease the expression of genes or alter the association with its partners, similar to what has been shown for β1/2-Adrenergic receptor[6]. The other hypothesis is that variants located in 3’UTR regions might regulate protein expression at post-transcriptional level. Another hypothesis might be that common SNPs are markers of functional rare variants that are not covered by current genotyping strategies. Functional studies are therefore warranted to clarify the biologic roles of these SNPs.

## Conclusions

Our results showed that two common genetic variations (rs361508, and rs3814843) in TRDN and CALM1 were independent predictors of SCD in patients with CHF in Chinese Han population. Patients with CC genotype of rs3814843 also had increased the risk of all-cause death. These findings suggest that common variants in genes may contribute to the pathogenesis of SCD.

## Study Limitations

Several potential limitations, including lack of functional studies, must be addressed so as to guide future research. In addition, the choice among 71 SNPs was performed in a small study samples, the potential for false-negative results should be evaluated in future, larger replication efforts; investigation of other variants in larger samples and replication among diverse population cohorts independently might also be important.

## Supporting Information

S1 Table List of 71 SNPs representing 22 genes. They were outlined in detail including gene names, rs number, position, MAF in CHB, source of data, and prediction of function by FuncPred (http://snpinfo.niehs.nih.gov/snpinfo/snpfunc.htm) etc.
(XLSX)

S2 Table Primers of 20 SNPs representing 10 genes. Primers were designed with a combination of previous articles and AssayDesigner3.1.
(XLSX)

S3 Table Clinical characteristics of patients with CHF. AF, atrial fibrillation; CHF, chronic heart failure; LVEDD, left ventricular end-diastolic diameter; LVEF, left ventricular ejection fraction; NYHA, New York Heart Association; VT, ventricular tachycardia; SBP, systolic blood pressure; DBP, diastolic blood pressure. Values are presented as mean±SD or n (%).
(DOCX)

S4 Table Results from log-rank test. Three models including dominant model, recessive model an additive model were constructed for each variant. Hardy-Weinberg equilibrium (HWE) of all SNPs was evaluated with the use of the chi-square test with one degree of freedom.
(XLSX)

S5 Table Results using a survival Cox regression analysis. Three models including dominant model, recessive model an additive model were constructed for each variant. All SNPs were calculated using two models. Model 1: unadjusted model. Model 2: model 1+age, LVEF, and other factors, including New York Heart Association Functional class, hypertension, diabetes mellitus, etc.
(XLSX)
