# Supplementary material for: Common Variants in TRDN and CALM1 Are Associated with Risk of Sudden Cardiac Death in Chronic Heart Failure Patients in Chinese Han Population
Source: PLoS One. 2015 Jul 21;10(7):e0132459. doi: 10.1371/journal.pone.0132459 (PMC4510877; doi:10.1371/journal.pone.0132459)
Supplement: S3 Table — AF, atrial fibrillation; CHF, chronic heart failure; LVEDD, left ventricular end-diastolic diameter; LVEF, left ventricular ejection fraction; NYHA, New York Heart Association; VT, ventricular tachycardia; SBP, systolic blood pressure; DBP, diastolic blood pressure. Values are presented as mean±SD or n (%). (DOCX) [file pone.0132459.s003.docx]

**Table 3. Clinical characteristics of patients with CHF**

|  | Patients with CHF | | | | | | |
| --- | --- | --- | --- | --- | --- | --- | --- |
| Clinical characteristic | ALL DCM | | | ICM | | DCM | |
| Age, y | | 63.76±12.27 | 65.56±10.57 | | 60.21±14.44 | |  |
| Male, n (%) | | 1129(79.01) | 761(80.53) | | 368(76.03) | |  |
| NYHA functional class,  n (%) | |  |  | |  | |  |
| Ⅰ | | 0 | 0 | | 0 | |  |
| Ⅱ | | 583(40.80) | 519(54.92) | | 74(15.29) | |  |
| Ⅲ | | 506(35.41) | 284(30.53) | | 222(45.87) | |  |
| Ⅳ | | 320(22.39) | 142(15.03) | | 188(38.84) | |  |
| Hypertension, n (%) | | 673(47.10) | 546(57.78) | | 127(26.24) | |  |
| Hyperlipidemia, n (%) | | 333(23.30) | 281(29.74) | | 52(10.74) | |  |
| Diabetes mellitus, n (%) | | 336(23.51) | 259(53.51) | | 77(15.91) | |  |
| Body mass index (kg/m^2^) | | 24.85±3.93 | 25.12±3.65 | | 24.31±4.43 | |  |
| SBP(mmHg) | | 116.81±19.76 | 119.82±20.20 | | 110.93±17.44 | |  |
| DBP(mmHg) | | 72.93±12.32 | 73.91±12.22 | | 71.02±12.30 | |  |
| Hemodynamic parameters | |  |  | |  | |  |
| LVEF (%) | | 39.40±12.05 | 42.98±11.16 | | 32.42±10.57 | |  |
| LVEDD (mm) | | 60.82±10.91 | 57.11±9.53 | | 68.04±9.76 | |  |
| Heart rate (beats/min) | | 77.14±15.96 | 75.08±14.42 | | 81.16±17.94 | |  |
| Nonsustained AF on Holter, n (%) | | 202(14.14) | 105(11.11) | | 97(20.04) | |  |
| Nonsustained VT on Holter, n (%) | | 110(7.70) | 66(6.98) | | 44(9.09) | |  |

AF, atrial fibrillation; CHF, chronic heart failure; LVEDD, left ventricular end-diastolic diameter; LVEF, left ventricular ejection fraction; NYHA, New York Heart Association; VT, ventricular tachycardia; SBP, systolic blood pressure; DBP, diastolic blood pressure.

Values are presented as mean±SD or n (%).
